# Monitoring phospholipid dynamics *in vivo* with a fluorescent dye octadecyl rhodamine B

**DOI:** 10.1247/csf.25126

**Published:** 2025-10-31

**Authors:** Li Hao, Caiyi Zhao, Kuninori Suzuki

**Affiliations:** 1 Department of Integrated Biosciences, Graduate School of Frontier Sciences, The University of Tokyo, Kashiwa, Chiba 277-8562, Japan; 2 Department of Molecular and Cell Biology, College of Letters and Science, University of California, Berkeley, CA 94720, USA; 3 Collaborative Research Institute for Innovative Microbiology, The University of Tokyo, Bunkyo-ku, Tokyo 113-8657, Japan; 4 Life Science Data Research Center, The University of Tokyo, Kashiwa, Chiba 277-8562, Japan

**Keywords:** autophagy, *in vivo* lipid dynamics, octadecyl rhodamine B (R18), phospholipase, phospholipid, vacuole, yeast

## Abstract

Phospholipids are major components of biological membranes. They play an essential role in intracellular signaling and organelle dynamics; however, the availability of suitable lipid-specific probes is limited, which has hindered studies on their spatial distribution and functional dynamics in living cells. Previously, we demonstrated that octadecyl rhodamine B chloride (R18) is transported to the endoplasmic reticulum via nonvesicular membrane transport. In this study, we showed that R18 is internalized in a phosphatidylethanolamine (PE)-dependent manner *in vivo*. The internalization of R18 in *Saccharomyces cerevisiae* is blocked in PE-deficient mutants, but restored by ethanolamine supplementation, which suggests strict PE dependence. Moreover, R18 delivered to vacuoles through autophagy was not terminally retained, but underwent Pep4- and Atg15-dependent export from the vacuoles. The exported R18 was then redirected to endosomes following prolonged autophagy. These results suggest that R18 may serve as an indicator of PE dynamics and vacuole–endosome lipid transport, which contributes to lipid homeostasis inside vacuoles.

## Graphical Abstract

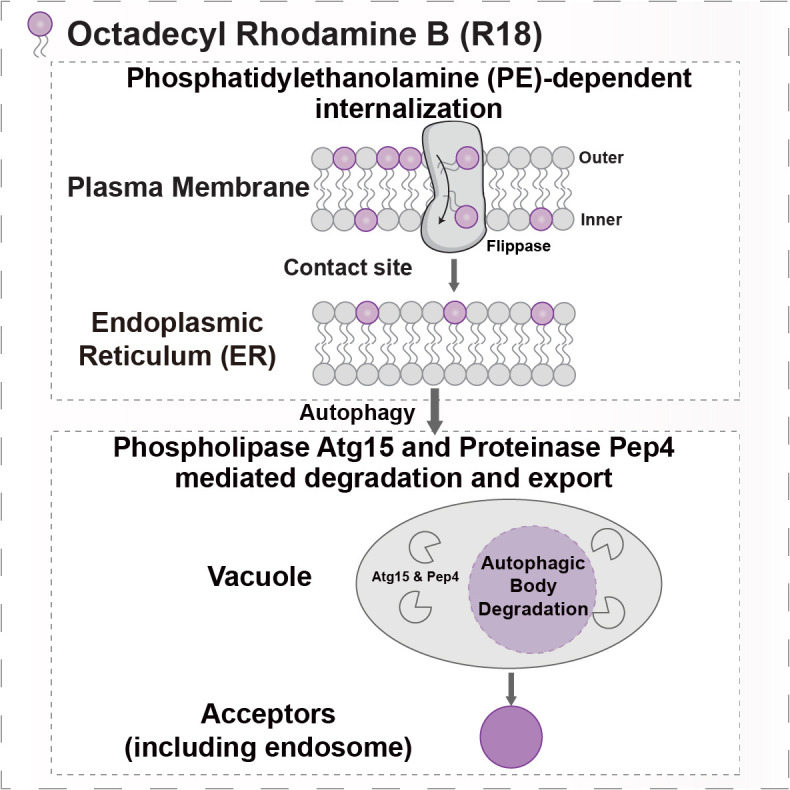

## Introduction

Cell membranes act as barriers to separate cells from the external environment or partition cells into distinct compartments. They consist of lipid bilayers containing phospholipids, sphingolipids, and cholesterol (Harayama and Riezman, 2018). Of these, phospholipids are highly diverse in structure and distribution. The composition of membrane lipids varies between organisms and cell types as well as across organelles, membranes, bilayer leaflets, and even subdomains within membranes (Hannich *et al.*, 2011; Yamashita *et al.*, 2014; Harayama *et al.*, 2014; Antonny *et al.*, 2015; Sezgin *et al.*, 2017; Lemmon, 2008). To maintain homeostasis, phospholipids are regulated by various enzymes, including flippases, floppases, and scramblases, to maintain their asymmetry within the membrane (Tanaka *et al.*, 2011; Sakuragi and Nagata, 2023). Phospholipids are highly dynamic between different organelles, and they may be transferred through the vesicular transport pathway via vesicles and the nonvesicular transport pathway via different lipid transfer proteins at membrane contact sites (Hanna *et al.*, 2023; Reinisch and Prinz, 2021; Wong *et al.*, 2019; Lev, 2010). There are numerous genetic disorders associated with lipid metabolism (Lamari *et al.*, 2013; Kuivenhoven and Hegele, 2014). Therefore, examining the biological significance of their diversity is of fundamental importance in biology and medicine.

The major phospholipids in eukaryotic membranes include phosphatidylcholine (PC), phosphatidylethanolamine (PE), phosphatidylserine (PS), phosphatidylinositol (PI), and phosphatidic acid (PA) (van Meer *et al.*, 2008). The development of intracellular probes has facilitated studies of PS (lactadherin C2 domain), PA (Spo20p-derived PA-binding domain), and phosphatidylinositol phosphates (PIPs) (Yeung *et al.*, 2008; Posor *et al.*, 2022; Kassas *et al.*, 2012; Tan and Finkel, 2022). PS contributes to the electrostatic environment of the cytoplasmic leaflet, anchors signaling proteins, and acts as an “eat-me” signal following externalization during apoptosis (Tsuchiya *et al.*, 2018; Middel *et al.*, 2016; Mioka *et al.*, 2021; Uchida *et al.*, 2011; Cho *et al.*, 2016). PIPs function as spatial and temporal membrane markers that regulate various cellular processes, including signaling, membrane trafficking, cytoskeletal organization, and autophagy (Michell, 2008; Balla, 2013). PA acts as a synthetic precursor of membrane phospholipids and a lipid signaling molecule that regulates membrane curvature, vesicle trafficking, and stress responses (Zhou *et al.*, 2024; Carman and Han, 2019); however, the role of PE remains poorly understood because of the lack of specific probes for *in vivo* tracking.

Previously, we used a screen to determine whether tracking dyes commonly used *in vitro* are suitable for *in vivo* studies. We found that octadecyl rhodamine B chloride (R18), a dye used for *in vitro* membrane fusion assays, but largely abandoned after 2000, was capable of staining the endoplasmic reticulum (ER) and autophagy-related structures in *Saccharomyces cerevisiae* and mammalian cells (Hao *et al.*, 2025; Hoekstra *et al.*, 1984; Hirata *et al.*, 2017). After the autophagosome fuses with a vacuole, R18 is transported to the vacuolar lumen. Interestingly, R18 is regulated by flippases at the plasma membrane (PM) and transferred through the nonvesicular transport pathway by shuttle- and bridge-type lipid transfer proteins (Hao *et al.*, 2025). However, it remains unclear the phospholipid dependence of R18 internalization and its subsequent fate after autophagic body disintegration remain elusive. In this study, we examined the internalization of R18 requires PE rather than PS, PC, or ergosterol using phospholipid-deficient yeast mutants and rescue assays. Moreover, we found that R18 is partially exported from the vacuole to the endosome in a process dependent on the vacuolar phospholipase Atg15 and the vacuolar proteinase Pep4, both of which are essential for the degradation of autophagic bodies in vacuoles (Watanabe *et al.*, 2023). Taken together, our results further suggest that R18 may serve as an indicator of PE dynamics.

## Results

### R18 intracellular localization is impaired in PE-deficient cells

**Fig. 1:**
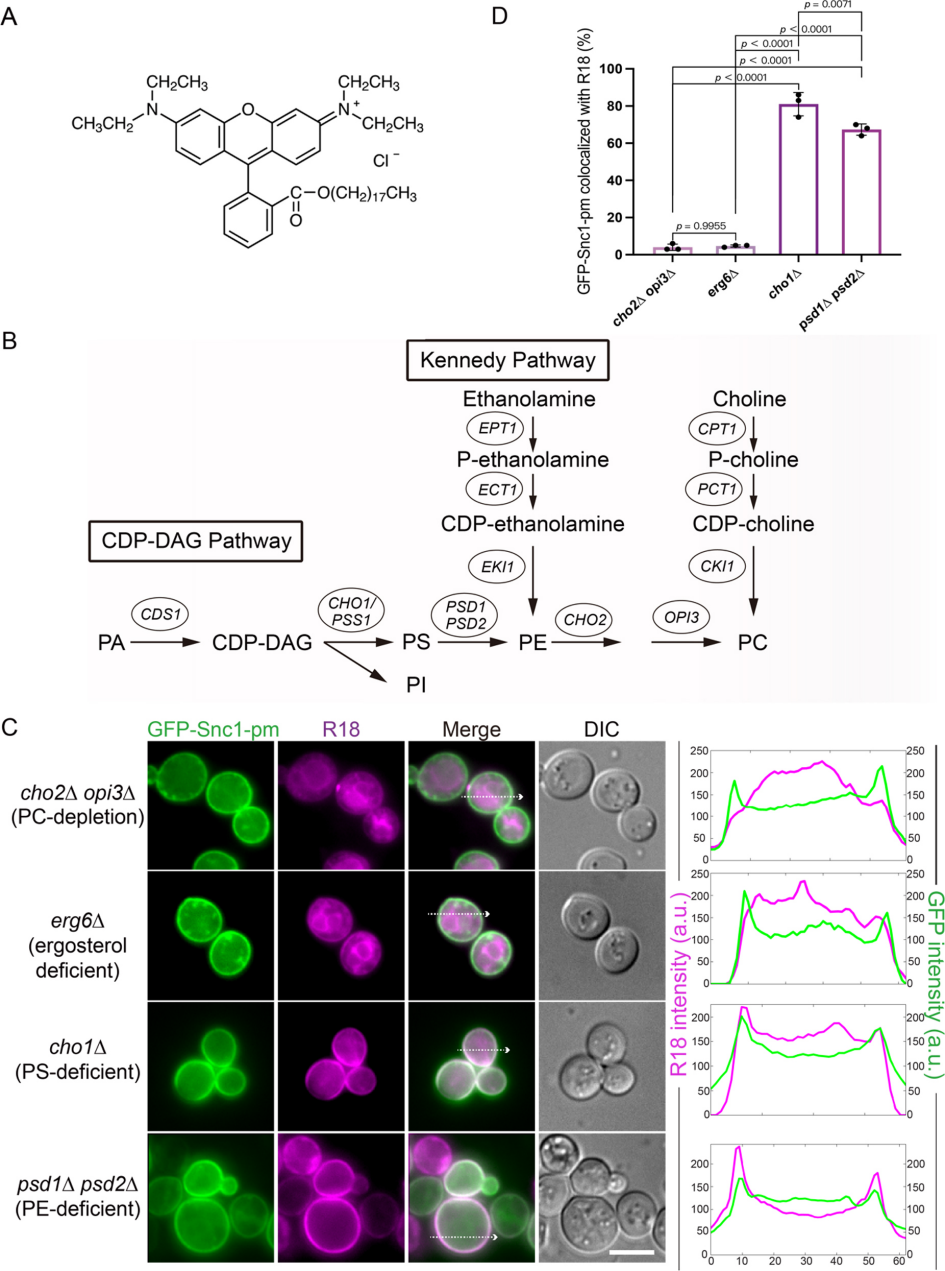
Phosphatidylethanolamine (PE) is required for the intracellular distribution of R18 (A) Chemical structure of R18, a lipophilic fluorescent dye with a C18 alkyl tail and a rhodamine head group (CAS RN: 65603-19-2). (B) Schematic representation of phospholipid biosynthesis pathways in yeast. PE and phosphatidylcholine (PC) are synthesized through the CDP–diacylglycerol (CDP–DAG) and Kennedy pathways. (C) Colocalization between GFP–Snc1–pm and R18 in phospholipid-deficient/depleted cells. The graphs represent the line profiles of the fluorescence intensity from the images on the left. Scale bar = 5 μm. DIC, differential interference contrast. (D) Percentage of colocalization calculated using 100 cells from 3 independent experiments in (C). Colocalization was judged visually based on the overlap of R18 and GFP signals at the cell periphery. Error bars represent SD. P-values were calculated using one-way ANOVA followed by Turkey HSD.

R18 is a lipophilic fluorescent dye comprising a long alkyl chain, which facilitates its insertion into a lipid bilayer (Fig. 1A). In wild-type cells, R18 exhibited a clear ER staining profile by biological pathways (Hao *et al.*, 2025; Hirata *et al.*, 2017). To determine whether R18 exhibits a dependence on specific phospholipid species, we analyzed R18 intracellular localization in phospholipid biosynthesis-deficient yeast mutants (Fig. 1B) (Carman and Henry, 1999; van Meer *et al.*, 2008). In the present study, we expressed green fluorescent protein (GFP)–Snc1–pm as a PM marker to assess the intracellular localization of R18. Normal R18 distribution patterns (ER) were observed in PC-depleted (*cho2*Δ *opi3*Δ) cells, which contained 6.6% ± 0.9% PC/total phospholipids (38.5% ± 0.8% in wild-type cells) as well as ergosterol-deficient (*erg6*Δ) cells (Hao *et al.*, 2025; Mioka *et al.*, 2021). R18 was internalized and labeled the ER with a minimum colocalization of GFP-Snc1-pm, indicating that PC and ergosterol are not required for its internalization (Fig. 1C). PS-deficient (*cho1*Δ) cells and PE-depleted (*psd1*Δ *psd2*Δ) cells containing 3.5% ± 1.1% PE/total phospholipids (18.8% ± 1.7% in wild-type cells) exhibited a blocking pattern of R18 in the PM co-localized with GFP–Snc1–pm (Fig. 1C and D) (Mioka *et al.*, 2021). Although the number of cells showing GFP–Snc1–pm colocalization appeared comparable, the intracellular fluorescence intensity of R18 tended to be lower in PE-deficient cells, suggesting that PE is more critically involved in the internalization and ER localization of R18.

Based on these observations, we assessed the putative PE-dependence of R18 *in vitro* using giant unilamellar vesicles (GUVs) with defined lipid composition. GUVs consisting of dioleoylphosphatidylcholine (DOPC) alone, or mixtures of 70% DOPC with 30% dioleoylphosphatidylserine (DOPS) or dioleoylphosphatidylethanolamine (DOPE), showed comparable levels of R18 fluorescence at the membrane and were similar under all conditions (Supplementary Fig. S1). Therefore, in contrast with the *in vivo* data, R18 showed no discernible dependence for PE under simplified reconstituted conditions, which suggests that additional features of the cellular milieu, such as lipid asymmetry, membrane curvature, or protein-mediated regulation, are required to manifest its selectivity.

### Defects in R18 localization are rescued by ethanolamine supplementation

**Fig. 2:**
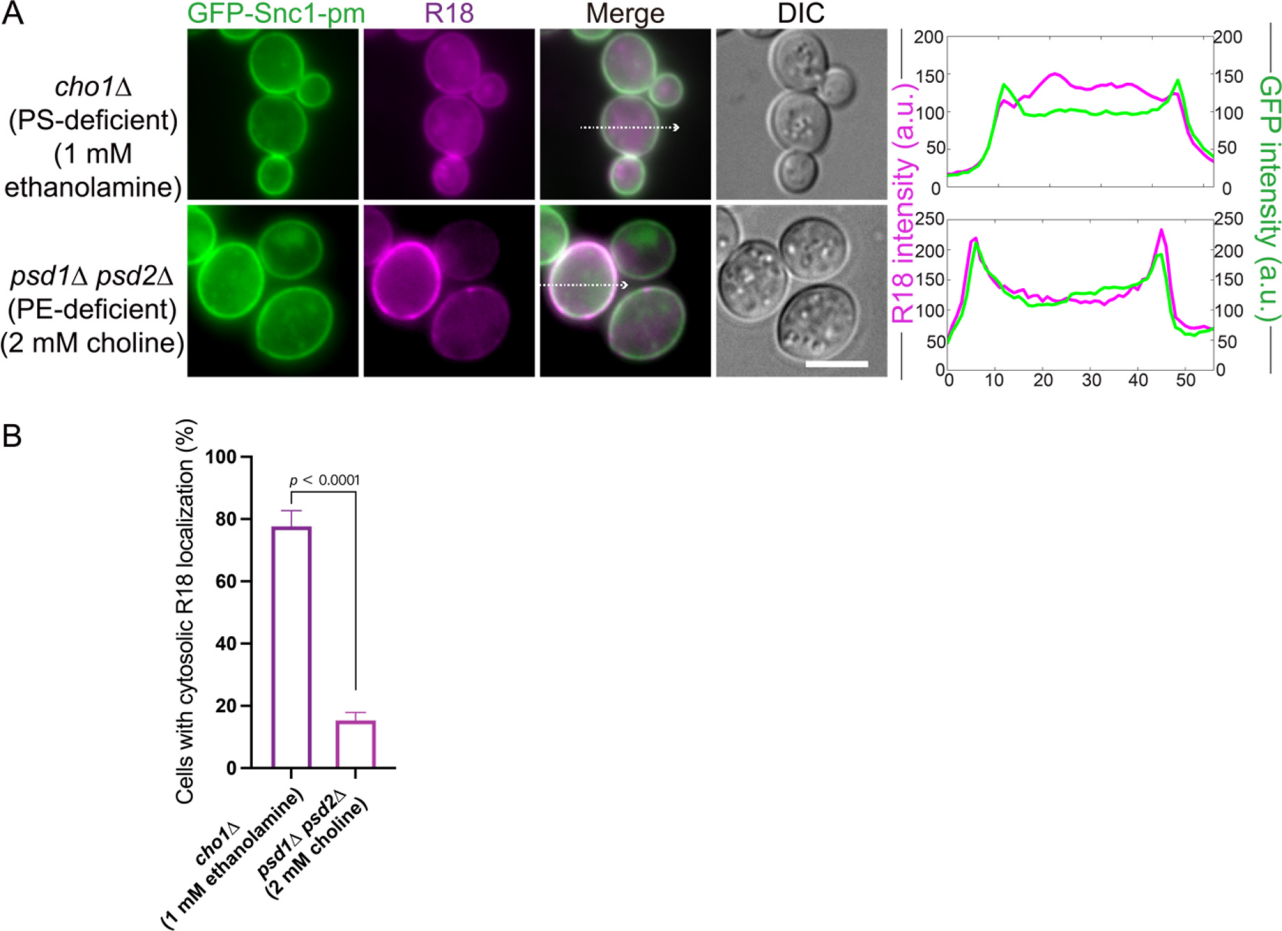
Ethanolamine supplementation restores R18 localization in phosphatidylserine (PS)-deficient cells (A) R18 localization in PS-deficient (*cho1*Δ) and phosphatidylethanolamine (PE)-deficient (*psd1*Δ *psd2*Δ) yeast cells following the addition of ethanolamine or choline. The graphs represent the line profiles of the fluorescence intensity from the images on the left. Scale bar = 5 μm. DIC, differential interference contrast. (B) Percentage of cells with cytosolic R18 localization calculated using 50 cells from 3 independent experiments in (A). Cytosolic localization was defined as visibly stronger fluorescence in the cytoplasm compared with the signal at the PM. Error bars represent SD. P-values were calculated using the Student’s *t*-test.

Since PE is downstream of PS synthesis, to determine whether the R18 localization defect results from PS or PE depletion, we conducted rescue experiments by supplementing the culture medium with ethanolamine. Ethanolamine promotes PE biosynthesis through the Kennedy pathway to mimic PS-only deficient cells (Fig. 1B) (Kennedy and Weiss, 1956). In PS-deficient *(cho1*∆) cells, ethanolamine supplementation restored R18 internalization, as evidenced by the decreased colocalization with GFP-Snc1-pm and increased intracellular fluorescence (Fig. 2A and B). Conversely, PC is downstream of PE synthesis. Therefore, in PE-deficient (*psd1*Δ *psd2*Δ) cells, the lack of PC likely causes an R18-staining deficiency; however, supplementation with choline to induce PC synthesis through the Kennedy pathway failed to restore R18 distribution (Fig. 2A and B), whereas R18 was strongly co-localized with GFP-Snc1-pm at the PM (Fig. 2A and B). These results indicate that PE is strongly implicated in R18 staining of the ER instead of the PM. Moreover, the recovery in *cho1*Δ cells suggests that even modest restoration of PE levels through the Kennedy pathway is sufficient for limited R18 internalization. Collectively, the results indicate that R18 exhibits PE-dependent dynamics *in vivo*.

### Pep4 and Atg15 are required for exporting the R18 signal from the vacuole to the endosome

**Fig. 3:**
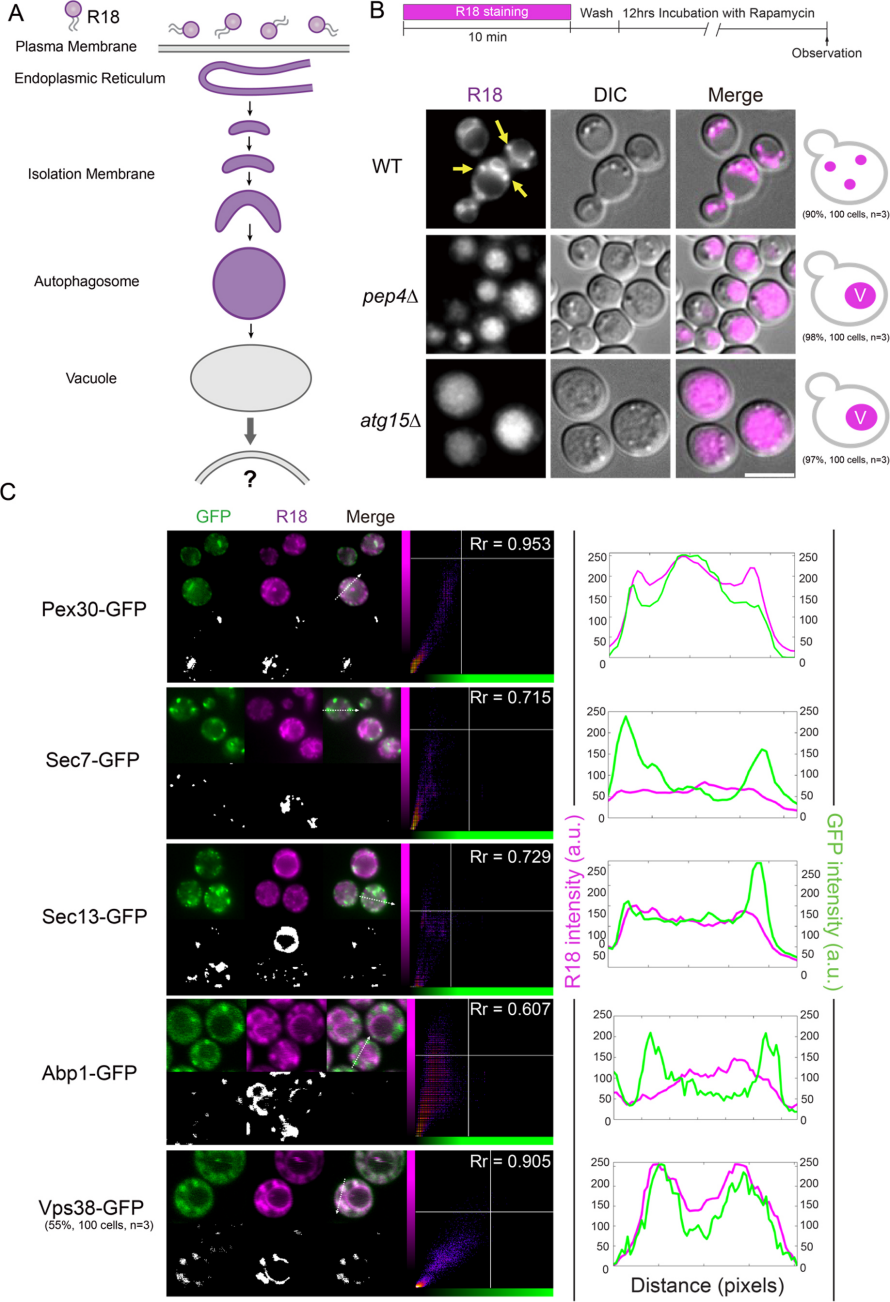
Vacuolar release of R18 labeling requires Pep4 and Atg15 (A) Schematic representation of the intracellular transport route of R18. After incorporation into the plasma membrane (PM), R18 is internalized and transported to the endoplasmic reticulum. R18 is then transferred to autophagy-related structures, including the isolation membrane, autophagosome, and autophagic bodies within the vacuole. (B) R18 localization in wild-type and autophagy-deficient yeast cells. The cells were stained with R18, washed, and incubated for 12 h in rapamycin-containing medium. The phenotypes of the yeast cells representing each pattern are illustrated on the right. Scale bar = 5 μm. (C) Colocalization analysis of R18 with GFP-tagged organelle markers after vacuolar export. Scatter density plots show fluorescence correlation, and line profiles indicate fluorescence intensity along the dotted lines. Scale bar, 5 μm.

Previously, we found that after flippase-mediated internalization of the PM, R18 is transported to the ER and transferred to the isolation membrane, which expands and closes into an autophagosome that eventually fuses with a vacuole (Fig. 3A) (Hao *et al.*, 2025). To identify the intracellular transfer route of R18, we tracked the R18 signal following vacuolar entry. After a 12-h incubation of R18-stained cells with rapamycin to induce autophagy in wild-type cells, the R18 signal was detected in large dot-like compartments outside of the vacuoles (Fig. 3B, arrows), suggesting its release from vacuoles into the downstream compartments. Both Pep4, a vacuolar proteinase, and Atg15, a vacuolar phospholipase, are required for the degradation of autophagic bodies in the vacuoles (Watanabe *et al.*, 2023). In *pep4*Δ and *atg15*Δ cells, R18 accumulated in the vacuolar lumen, which indicates that vacuolar protease activity (Pep4) and phospholipase-mediated membrane disruption (Atg15) are required for the efficient export of R18-derived signals from the vacuoles (Fig. 3B).

To determine the destination of the R18 signals, we performed quantitative colocalization analyses using GFP-tagged organelle markers. R18 displayed minimal overlap with the Golgi apparatus (Sec7-GFP), actin patches (Abp1-GFP), or ER exit sites (Sec13-GFP). Instead, both Pex30–GFP and Vps38–GFP exhibited relatively high Pearson’s correlation coefficients (Rr = 0.953 and 0.905, respectively) (Fig. 3C). Considering that R18 appeared as punctate structures during its export from vacuoles, we performed a detailed line-scan analysis to assess the spatial overlap. Although the overall correlation with Pex30–GFP was high, their signals did not coincide at discrete puncta. In contrast, Vps38–GFP displayed clear punctate colocalization with R18, indicating that the exported R18-containing structures are associated with endosomes rather than peroxisomes. Because only about half of the observed cells displayed such prominent punctate colocalization, we cannot exclude the possibility that other compartments, including the ER, may also serve as partial destinations of the exported R18 signals. Nevertheless, these results suggest that R18 delivered to the vacuoles through autophagy is exported in a Pep4- and Atg15-dependent manner and subsequently redirected to the endosomes.

## Discussion

R18 was originally developed as a dye for membrane fusion assays. Although its use declined (Hoekstra *et al.*, 1984; Chernomordik *et al.*, 1997), our previous studies revealed that it is actively internalized by flippases, transported by lipid transfer proteins, and labels ER and autophagy-related membranes (Hao *et al.*, 2025; Hirata *et al.*, 2017). In the present study, we extended these observations by showing that R18 localization is strictly dependent on PE in yeast cells. In addition, we found that R18 delivered to vacuoles through autophagy can undergo Pep4- and Atg15-dependent export from vacuoles and redistribution to endosomes. This suggests that autophagy-derived phospholipids enter a vacuole-to-endosome recycling pathway.

R18 failed to internalize in PE- and PS-deficient yeast cells *in vivo* and accumulated on the PM (Fig. 1). This phenotype was partially restored by ethanolamine supplementation through the Kennedy pathway, which suggests that R18 internalization is PE-dependent (Fig. 2). Taken together, these results indicate the robust dependence of R18 on PE-containing membranes. Thus, instead of a PE-specific probe, R18 has potential as an indicator of PE dynamics in living cells. For example, because R18 is transferable by lipid transfer proteins, its dependence of PE may enable it to function as a traceable marker for lipid transfer activity to evaluate the lipid transfer direction at different membrane contact sites (Hao *et al.*, 2025). Moreover, R18 may be used in living cells to monitor lipid transport in real time.

An important question that arises from these observations is why the internalization of R18 depends more on PE. *In vitro* assays using GUVs with defined lipid compositions failed to recapitulate this dependence, as R18 exhibited comparable labeling across PC-, PS-, and PE-containing membranes (Supplementary Fig. S1). This discrepancy emphasizes the limitations of simplified reconstitution systems and suggests that the selectivity of R18 has emerged only in a cellular context. Factors, such as lipid asymmetry, negative curvature, acyl chain diversity, and protein-mediated regulation, may all contribute. Consistent with this idea, we previously demonstrated that R18 is selectively transported by the bridge-type lipid transport protein Atg2 to autophagy-related membranes, but not to mitochondria or other compartments. This indicates that its *in vivo* selective staining pattern reflects regulated transport, rather than intrinsic lipid affinity. To examine this further, we constructed a predicted complex model of the lipid transfer protein Atg2, with R18, DOPE, and POPC, using AlphaFold3 (Supplementary Fig. S2) (Abramson *et al.*, 2024). R18 was accommodated alongside DOPE and POPC, suggesting physical compatibility within the lipid-binding cavity; however, direct molecular interactions between R18 and the phospholipid headgroups were not observed. Therefore, we considered the possibility that electrostatic complementarity between the rhodamine moiety of R18 and the zwitterionic headgroup of PE underlies the R18 internalization dependence. Further structural and biophysical studies are needed to elucidate the molecular basis of the PE dependence of R18 staining.

We extended our observations to the post-vacuole stage after autophagosomes were fused with vacuoles. To determine whether R18 is released from vacuoles, we prolonged the incubation period to 12 hours. R18 delivered to vacuoles was not terminally retained, but underwent Pep4- and Atg15-dependent release, and was subsequently redistributed to the endosomes (Fig. 3). These results suggest that autophagy may not only deliver membrane material to vacuoles for degradation, but it also contributes to recycling that channels lipid back into the endosomal system. Such recycling may balance degradation with reuse and sustain membrane homeostasis under nutrient stress. Because phospholipids are very hydrophobic, they cannot freely diffuse through the aqueous cytosol to reach the endosomes. How phospholipids are exported from vacuoles and subsequently transported to endosomal compartments remains unclear and will be the subject of future studies. Whether analogous pathways operate in higher eukaryotes and how they intersect with metabolic adaptation and organelle biogenesis are also important questions for future studies.

## Methods

### Yeast strains and media

The *S. cerevisiae* strains are listed in Supplementary Table S1. The cells were cultured in YPD (1% Bacto^TM^ yeast extract, 2% Bacto^TM^ peptone, and 2% glucose), SDCA (0.17% Difco^TM^ yeast nitrogen base w/o amino acids and ammonium sulfate, 0.5% ammonium sulfate, 0.5% Bacto^TM^ casamino acids, and 2% glucose), containing the appropriate nutrients. Standard protocols were used for yeast manipulation (Kaiser *et al.*, 1994).

### Fluorescence microscopy

The cells were grown overnight, diluted, and cultured in SDCA medium supplemented with the appropriate nutrients until log phase (~2 × 10^7^ cells/mL; OD_600_ = ~1). For R18 staining, the cells were incubated with R18 (Invitrogen) at 5 μg/mL (from a 1 mg/mL stock dissolved in dimethyl sulfoxide) for 10 min in a nutrient-rich medium at 30°C. Next, the cells were washed three times with fresh medium. Fluorescence imaging was conducted using an IX83 inverted system microscope (Olympus) equipped with a UPlanSApo100× oil-immersion lens (1.40 NA; Olympus) and a CoolSNAP HQ CCD camera (Nippon Roper). U-HGLGPS (Olympus). A mercury light source system was used to excite the fluorescent proteins. U-FGFP and U-FRFP filter sets (Olympus) were used for GFP and R18 visualization, respectively. The resulting images were acquired using MetaVue imaging software (Molecular Devices). Samples were prepared on 76 × 26-mm glass slides (S1225; Matsunami) with 18 × 18-mm glass coverslips (No. 1-S; Matsunami).

### Preparation of GUVs

DOPC, DOPS, and DOPE were obtained from Avanti Polar Lipids. A natural swelling method was used to produce the GUVs (Yamazaki, 2008; Fujioka *et al.*, 2020; Yamasaki *et al.*, 2020). Briefly, 200 μL of 1 mM phospholipid mixture consisting of DOPC, DOPS, and DOPE in chloroform was dried under nitrogen gas to produce a lipid film in a 5-mL glass vial. The lipid film was left overnight under vacuum to remove the remaining chloroform. Next, it was prehydrated with 20 μL of water at 60°C for 7 min, and 1.0 mL of HEPES buffer (20 mM HEPES–NaOH, pH 7.0, 150 mM NaCl, and 1 mM EGTA), containing 0.1 M sucrose, was added. The GUVs were spontaneously produced in solution by incubating at 60°C for 2–3 h and then gently cooled at room temperature.

### Confocal fluorescence microscopy

A custom microchamber was prepared by placing two 3-mm-thick, bar-shaped silicon-rubber spacers parallel to one another between a cover slip (Muto Pure Chemicals) and a glass slide (Matsunami Glass). They were coated with 0.1% (w/v) bovine serum albumin (BSA, Wako) in HEPES buffer supplemented with 0.1 M glucose to avoid the adhesion of GUVs to the glass surface. After BSA coating, 100 μL of the GUV solution was diluted with 900 μL of HEPES buffer containing 0.1 M glucose in the microchamber. Time-lapse confocal observations of the GUVs were performed using a laser-scanning confocal microscope FV3000RS (Olympus) with FV31S-SW software at room temperature, as previously described (Fujioka *et al.*, 2020; Yamasaki *et al.*, 2020). The images were analyzed using ImageJ software (https://fiji.sc/).

### Colocalization analysis

Fluorescence colocalization between R18 and GFP-Snc1-pm was judged visually based on the overlap of R18 and GFP signals at the cell periphery. Cytosolic localization was defined as visibly stronger fluorescence in the cytoplasm compared with the signal at the PM.

Fluorescence colocalization between R18 and GFP-tagged markers was quantified using the JACoP (Just Another Colocalization Plugin) in FIJI/ImageJ (Bolte and Cordelières, 2006). Pearson’s correlation coefficients (Rr) were calculated after thresholding. Scatter density plots were generated to visualize the pixel intensity correlation between the two fluorescence channels. Line profiles of fluorescence intensity were obtained using the “RGB Profile Plot” along manually defined regions of interest (ROIs).

### Ethanolamine and choline supplementation assay

PS-deficient (*cho1*Δ) and PE-deficient (*psd1*Δ *psd2*Δ) cells were cultured overnight at 30°C in SDCA medium containing the appropriate nutrients. The cultures were diluted and grown until log phase (~2 × 10^7^ cells/mL; OD_600_ ≈ 1.0). For ethanolamine supplementation, 1 mM ethanolamine hydrochloride (Fujifilm) was added to the *cho1*Δ cells. For choline supplementation, 2 mM choline chloride (Fujifilm) was added to the medium of the *psd1*Δ *psd2*Δ cells. The cells were stained with R18 and imaged by fluorescence microscopy to assess R18 localization.

### Statistical analysis

All statistical analyses were performed using GraphPad Prism software (version 9.5.1).

## Conflict of Interest

The authors declare that this study was conducted in the absence of any commercial or financial relationships that could be construed as potential conflicts of interest.

## Data availability Statement

The datasets presented in this study and the original data are available from the corresponding author upon reasonable request.

## Author Contributions

KS supervised the project. LH and KS conceptualized the study and designed the experiments. CZ and LH performed the R18 export and colocalization experiments. LH performed other experiments and data analysis. LH wrote the original manuscript. LH and KS revised the manuscript.

## Abbreviations

CDP-DAG: cytidine diphosphate–diacylglycerol
DOPE: 1,2-dioleoyl-sn-glycero-3-phosphoethanolamine
DOPC: 1,2-dioleoyl-sn-glycero-3-phosphocholine
DOPS: 1,2-dioleoyl-sn-glycero-3-phospho-L-serine
ER: endoplasmic reticulum
GFP: green fluorescent protein
GUV: giant unilamellar vesicle
HEPES: 4-(2-hydroxyethyl)-1-piperazineethanesulfonic acid
PA: phosphatidic acid
PC: phosphatidylcholine
PE: phosphatidylethanolamine
PI: phosphatidylinositol
PIP: phosphatidylinositol phosphate
PM: plasma membrane
POPC: 1-palmitoyl-2-oleoyl-glycero-3-phosphocholine
PS: phosphatidylserine
R18: octadecyl rhodamine B chloride
WT: wild type
