# Supplementary material for: Monitoring phospholipid dynamics in vivo with a fluorescent dye octadecyl rhodamine B
Source: Cell Struct Funct. 2025 Oct 31;50(2):213–21. doi: 10.1247/csf.25126 (PMC12967523; doi:10.1247/csf.25126)
Supplement: Supplementary file 1 — Supplementary Materials [file csf_50_25126_1.zip › 50_25126_SupplementaryMaterials.docx]

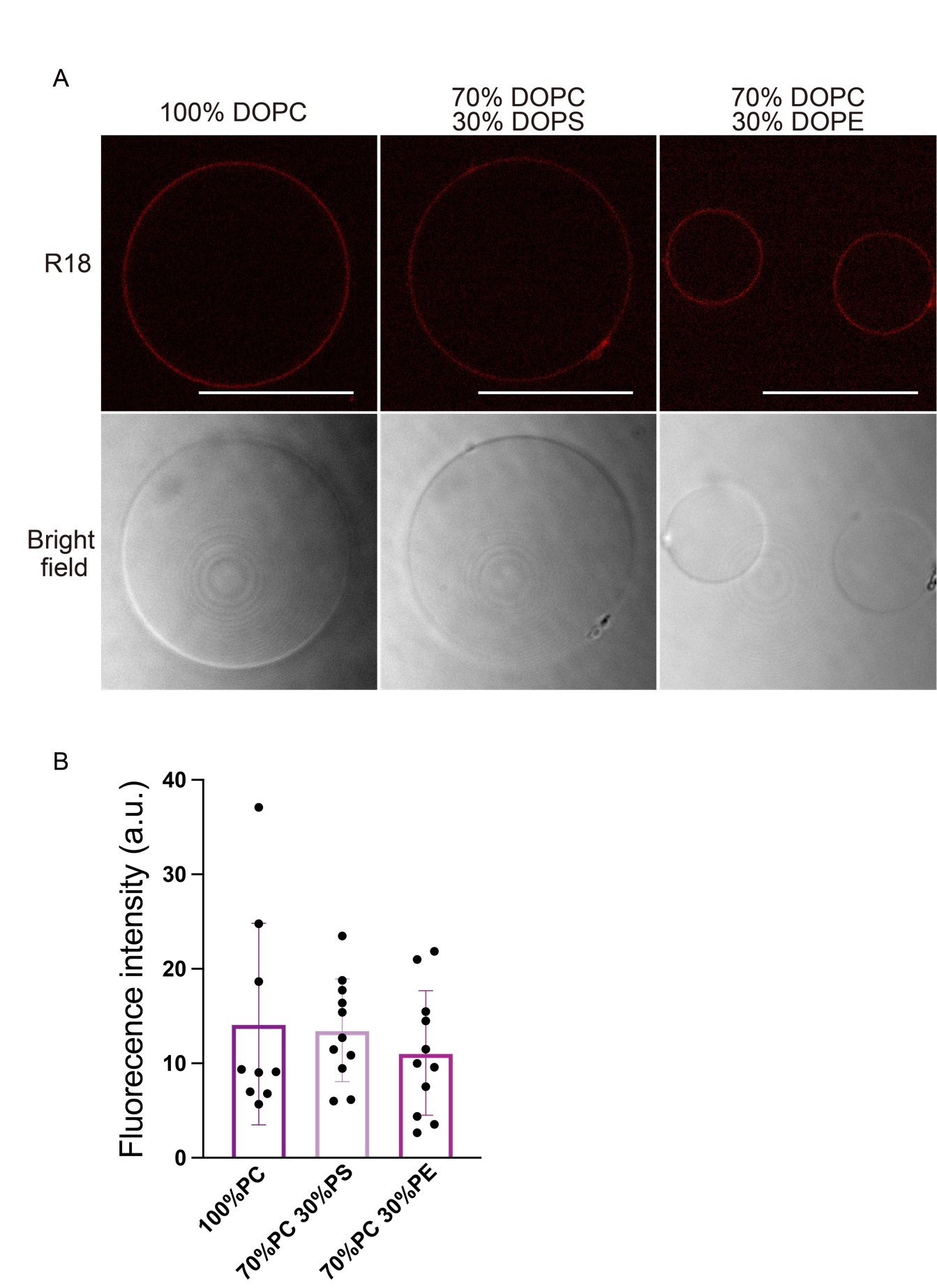


**Supplementary Fig. S1.** R18 shows no significant preference among different giant unilamellar vesicles (GUV) lipid compositions in vitro

(A) R18 staining of GUVs consisting of different phospholipid mixtures. Scale bar = 20 µm.

(B) Quantification of membrane-associated R18 fluorescence intensities in GUVs shown in (A). Bars represent mean ± SD from independent GUV preparations. No significant differences were observed among the groups (two-sided Student’s *t*-test; ns).


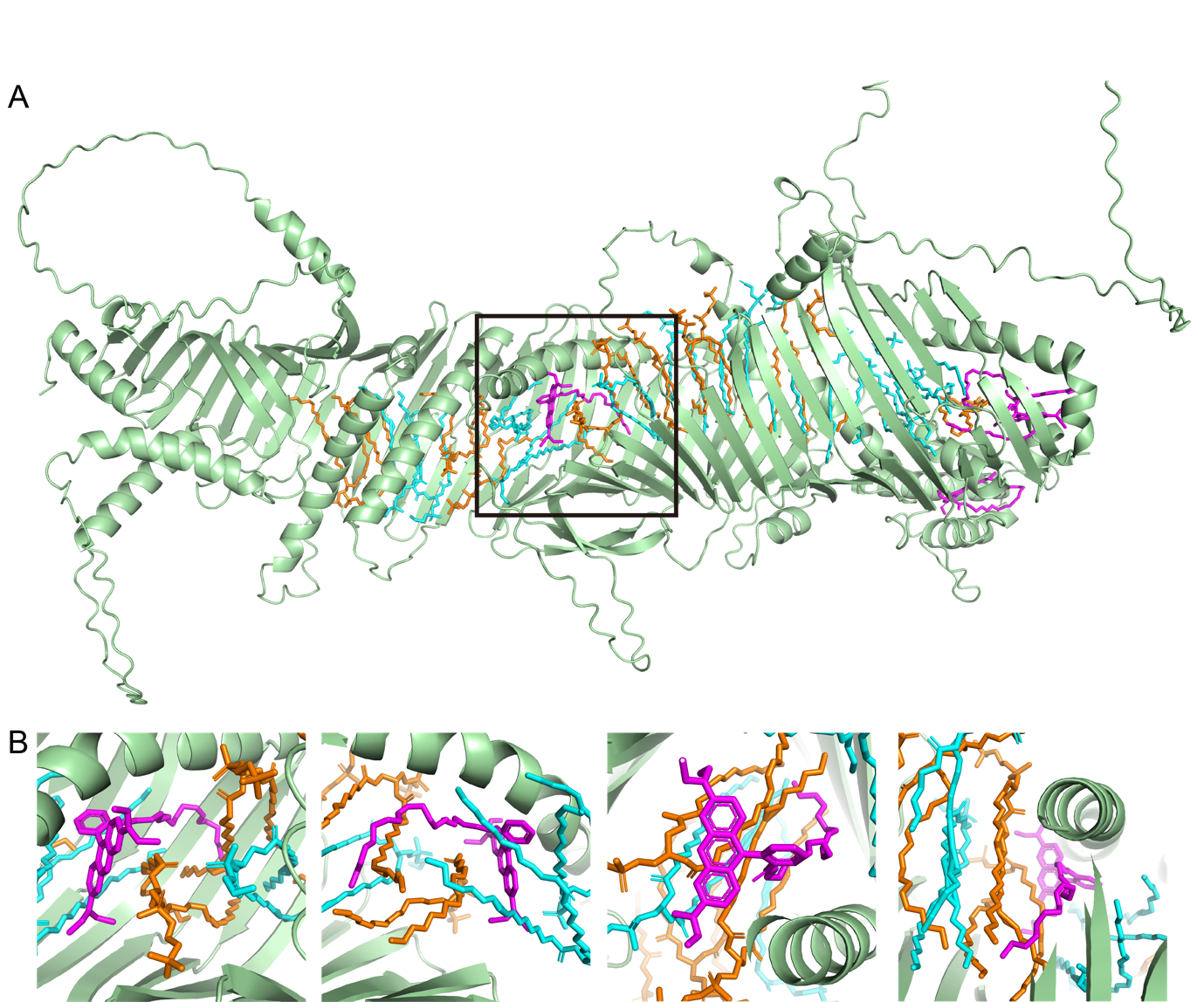


**Supplementary Fig. S2. Predicted structural model of R18 and phospholipids within a lipid transfer protein generated using AlphaFold3**

The structure shows a lipid transfer protein model bound to 12 molecules of phosphatidylcholine (POPC; orange), 12 molecules of phosphatidylethanolamine (DOPE; cyan), and 4 molecules of octadecyl rhodamine B chloride (R18; magenta). (A) The overall predicted structure with a boxed region indicating the central lipid-binding cavity. (B) present magnified views of this region.

**Supplementary Table S1.** *S. cerevisiae* strains used in this study.

| Strain^a^ | Mat | Genotype | Source |
| --- | --- | --- | --- |
| BY4741 | α | *his3Δ1 leu2Δ0 met15Δ0 ura3Δ0* | (Brachmann et al., 1998) |
| YKT38 | a | *ura3-52 his3-200 trp1-63 leu2-1 lys2-801* | (Mioka et al., 2021) |
| YKT2008 | a | YKT38 *cho1Δ::hphMX4 LEU2::GFP-SNC1-pm TRP1* | (Mioka et al., 2021) |
| YKT2241 | a | YKT38 *cho2Δ::HIS3MX6 opi3Δ::kanMX6 LEU2::GFP-Snc1-pm* | (Mioka et al., 2021) |
| YKT2242 | α | YKT38 *psd1Δ::kanMX6 psd2Δ::HIS3MX6 LEU2::GFP-Snc1-pm* | (Mioka et al., 2021) |
| YKT2245 | α | YKT38 *erg6Δ::kanMX6 LEU2::GFP-SNC1-pm TRP1* | (Mioka et al., 2021) |
| YHL2 | α | SEY6210 *atg15*Δ*::hphNT1* | (Hao et al., 2025) |
| YHL3 | α | SEY6210 *pep4*Δ*::LEU2* | (Hao et al., 2025) |
| Pex30-GFP | α | BY4741 *pex30Δ::PEX30-GFP:HIS3MX6* | (Huh et al., 2003) |
| Sec7-GFP | α | BY4741 *sec7*Δ*::SEC7-GFP:HIS3MX6* | (Huh et al., 2003) |
| Sec13-GFP | α | BY4741 *sec13*Δ*::SEC13-GFP:HIS3MX6* | (Huh et al., 2003) |
| Abp1-GFP | α | BY4741 *abp1*Δ*::ABP1-GFP:HIS3MX6* | (Huh et al., 2003) |
| Vps38-GFP | α | BY4741 *vps38*Δ*::VPS38-GFP:HIS3MX6* | (Huh et al., 2003) |

^a^: YKT strains are isogenic derivatives of YEF473 (S12).

Brachmann, C.B., A. Davies, G.J. Cost, E. Caputo, J. Li, P. Hieter, and J.D. Boeke. 1998. Designer deletion strains derived from *Saccharomyces cerevisiae* S288C: a useful set of strains and plasmids for PCR-mediated gene disruption and other applications. *Yeast*. **14**:115–132.

Giaever, G., and C. Nislow. 2014. The yeast deletion collection: a decade of functional genomics. *Genetics*. **197**:451–465.

Hao, L., T. Midorikawa, Y. Ogasawara, Y. Hama, H. Lang, N.N. Noda, and K. Suzuki. 2025. Reversible one-way lipid transfer at ER–autophagosome membrane contact sites via bridge-like lipid transfer protein Atg2. *bioRxiv*. 2025.03.27.645728.

Huh, W.K., J.V. Falvo, L.C. Gerke, A.S. Carroll, R.W. Howson, J.S. Weissman, and E.K. O’Shea. 2003. Global analysis of protein localization in budding yeast. *Nature*. **425**:686–691.

Mioka, T., T. Guo, S. Wang, T. Tsuji, T. Kishimoto, T. Fujimoto, and K. Tanaka. 2021. Characterization of micron-scale protein-depleted plasma membrane domains in phosphatidylserine-deficient yeast cells. *J Cell Sci.* **135**:jcs256529.
